# Role of Digital Technology in Transforming Organizational Competencies Influencing Green Economy: Moderating Role of Product Knowledge Hiding

**DOI:** 10.3389/fpsyg.2021.792550

**Published:** 2021-12-23

**Authors:** Haoran Bai

**Affiliations:** School of Humanities, Southeast University, Nanjing, China

**Keywords:** digital technology, misinformation, entertainment, education, transparency, centralization, green economy, knowledge hiding

## Abstract

Digital technology has gained momentum in the recent decade, with its relationships with digital entrepreneurship, digital economies, digital social interaction, green economies, etc. These have changed the perspective of business and hence digitalized the strategic policies through blockchains. The current study aims to identify such benefits that have changed the day-to-day life processes and procedures for carrying out different tasks due to the convenience of adopting digital technology. Those benefits have been classified as transparency, centralization, and access to new markets for the organizations considering their consequences, especially when using digital technology. When processes are taking place online, there are fair chances of hiding knowledge about certain products or procedures to gain particular benefits. Hence, this study has considered the moderating role of product knowledge hiding while interacting online. This study is a quantitative post-positivist cross-sectional study that has followed a survey technique for data collection. The population used in this study is the managerial staff of the telecom sector in the mainland in China. The sample size used in this study is 358. The software used in this study is Smart-PLS 3.3. The technique used in this study for data analysis is structural equation modeling with measurement modeling. The findings of this study show that digital technology has led to many benefits for organizations like centralization, access to the new markets, and transparency, which have been made possible remotely only because of the use of digital technology in business operations. However, the moderating role of product knowledge hiding has been found significant only for transparency. This research paper highlights the important benefits of the use of technological use in the corporate world. Also, it contributes to expanding the network of knowledge hiding, addressing the moderation of product knowledge hiding, and extending the known consequences of digital technology influencing knowledge hiding.

## Introduction

Digital technology has aided in the shift to a green economy by conserving resources and energy (reducing environmental consequences) as well as promoting the use of clean technology and products with reusable goods (push impacts; Kivimaa et al., 2021). The Internet of Things and the sharing economy are two specific examples of the impact of digital technologies on the transition to a green economy. The beneficial impact is linked to using technology in the manufacturing process (cutting energy, lowering the number of supplies used), the substitution of virtual goods for physical goods (e-commerce instead of office operations, e-books rather than physical books), and resource-saving sensors (turning off the light in the absence of people, stopping irrigation after the soil is saturated with water). Some researchers added to the benefits of digital technologies by claiming that they save assets not only in the manufacturing process (*via* the use of detectors and monitors) but also in the supply channel through the closing of kiosks and the move to internet commerce (Su et al., 2021). Digital technology can start driving green economies in different ways: by reducing the direct environmental impact of ICT production, distribution, operation, and disposal, by reducing toxic materials and waste disposal, by enhancing the speed of production, allocation, and consumption of goods and services in the economy and society as a whole, and by reducing power consumption and decreasing the amount of assets, substituting resources.

In order to save energy and resources, products and services are being shared, and it has only become feasible because of digital technologies, which manage the presence of unused capabilities that connect merchants and buyers on specialized digital platforms (Curtis, 2021). Many states and international organizations have already expressed their gratitude for the advantages of digital technologies for transitioning to a more sustainable future. The clean economy documents from the United Nations also confirm this. The EU launched a program for the transformation to a green knowledge society in 2015, which states that it intends to encourage the use of technology for emission reduction in all sectors and assure the use of technology for further sustainability and company behavior models. In light of the world’s worsening environmental situation and high levels of greenhouse gas emissions, researchers are increasingly focusing on producing renewable energy to replace conventional fuels. These systems allow businesses to save more and spend less and do so at the lowest possible cost, all through common platforms rather than a patchwork of technology systems.

The development of renewable energy sources is a priority for both developed and developing countries. The United States, European countries, China, India, and Japan are leading investors in renewable energy. Issues are impeding the growth of renewable energy sources. Some of these issues are significant, such as the current lack of competitiveness of renewable energy in Russia, difficulties in purchasing equipment and technologies owing to sanctions, a fall in energy use due to the crisis, and the availability of inexpensive renewable sources (oil, gas, and coal). It is feasible to use digital technology to solve the problem of non-competitiveness, which is one of the most serious issues. Global technology businesses and industrial behemoths have reached the point where they must pursue a platform strategy after a succession of pilot initiatives in the realm of digital solutions. When it comes to establishing Internet of Things platforms, it is crucial to consider the platform’s core features, such as data collecting, storage, processing, and modeling, and the business applications deployed as platform solutions to produce value.

There are a variety of assistance structures in place in various nations to help industrial enterprises shift to more sustainable resources. Dematerialization is the instant positive environmental effect of digitization. Transitioning to electronic document control, digital services and products in trade, financial services, and organizational realms, and replacing physical logistics flows with remote means of communication based on digital technologies (e-mail and bulletin boards, video conferencing, electronic exchanges, e-government services, and so on) have resulted in a decrease in time, economic, and information assets retrieved from the natural environment. Resultantly, the amount of garbage generated by businesses, organizations, and end-users lowers, reducing the anthropogenic strain on ecosystems dramatically.

Increasing the market for smart gadgets and digital services leads to an increase in energy usage, greenhouse gases, and the buildup of electronic waste, which has negative environmental consequences. Price gouging and tactics to maximize monopolistic quasi-rent from pseudo-innovation, when marketing practices that encourage overconsumption for prestige motives replace real research and development, increase these negative results.

With the benefits of using digital technology to reinforce the green economy, knowledge hiding behaviors also have a significant role in moderating the factors involved. A new cyber risk is product knowledge hiding. Misinformation, disinformation, knowledge hiding, and false information are all examples. While much has been said about the implications of product knowledge hiding, disinformation, and false information on the political process, the consequences of false information on businesses have received far less attention and have been considered to be inadequately researched (Petratos, 2021; Young, 2021). This research was based on certain objectives finding out the benefits of using digital technologies to reinforce the green economy. Many factors including the moderating factor of product knowledge hiding, were studied and analyzed in this article. The objectives of the research were to estimate the effect of use of digital technologies for organizational benefits such as transparency, access to new markets, and centralization, and to explore the moderating role of product knowledge hiding on dependent variables, i.e., centralization, access to new markets, and transparency in organizations.

## Review of Literature

### Impact of Digital Technology on the Access to New Markets

Academic institutions must adjust their structures to embrace digitalization and sustainable economic approaches in an attempt to reach all of the requirements of modern labor innovation and consumer technologies in terms of access to awareness and knowledge (Dahwan and Raju, 2021). The educational sector, particularly at the university level, has various organizational structures compared to the economic, banking, and administration of employee benefits. In the past few years, but particularly in recent years, the terms digitalization and disruptive technologies have been the most widely used terms (Gallaher et al., 2021; Josephy and Marko, 2021). Digitalization, which is used to characterize the password-protected migration of commercial enterprises and businesses, has a plethora of interpretations.

The country’s economic digital shift has now become apparent (Mihelj and Jiménez-Martínez, 2021). From this standpoint, determining the overall impact of digital marketing on the development of new business models as well as human and organizational behaviors becomes critical (Kazancoglu et al., 2021). In light of the contemporary digital problems, it is also critical to establish economic and business performance factors. Technology innovations create new requirements for the processing of information in businesses, allowing them to change the processes and operations. Communication technology advancements aided organizational reaction to technological developments, therefore digital organizations should generate new capacities to foresee and adapt to changing developments in their business ecosystems (Hai et al., 2021). Inside the case of information technology development’s revolutionary trends, pervasive computing produces an ecosystem in which the provision of infrastructure, computers, sensing, and digital communication technologies are accessible everywhere. Furthermore, emerging digitalization establishes a new type of interconnected electronics in which sophisticated techniques automatically monitor and control satellite technology and mobile devices, including intelligent machinery (Wu et al., 2021).

Digital firms must build operational technologies simultaneously as they develop information technology (Volberda et al., 2021). As a result, a converging process is required and heavily reliant on harmonizing communication and operational technology plans. Keeping in view the literature available, the following hypothesis was devised.

*H_1_: Use of digital technology has an impact on the access of new markets by organizations.*

### Centralization of System With the Use of Digital Technology

The question about balancing the political legitimacy of something like the central government and public authorities in environmental protection has long been a source of heated discussion (Eriksson et al., 2021). Because both economic and environmental regulations are governmental regulatory instruments, parliamentary sovereignty and processes must be addressed when determining the amount to which local governments should be empowered (Overdevest and Zeitlin, 2018). When faced with the conflicting pressures of environmental conservation and preserving economic growth, the central government must find a balance (Nawaz et al., 2019). Our study analyzes the effects of functional and divisional administration on the effectiveness of environmental regulation using a two principal model.

Awareness and developing business strategy solutions require a clear understanding of the technology, commercial, and policy components of environmental issues (Vahdat, 2021). Whereas the influence of emerging regional and international carbon control regimes on firms has been researched for some time, management literature on the physiological consequences of climate change for business transformation has received very little attention (Tao et al., 2021). Global warming has ramifications on the environment, society, politics, and economy. The frequency and severity of extreme climate, disasters, droughts, disappearing Arctic glaciers, and rising sea levels are just a few of the major issues caused by global warming, all of which have significant implications for global trade.

Developing-country businesses are particularly vulnerable. Economic adaptation will result from proprietors changing their resource distribution related to the current ability to create value and pricing changes in the new environment that takes the demand, availability, and trade into account (Freitas Gomes et al., 2021). If the advantages of minimizing informational asymmetries outweigh the increased agency expenditures, centralization in the institutional framework will become more useful to the centralized administration; alternatively, effective centralized governance will be preferable. Enterprises face increased risks as a result of climate change. When it comes to the link between climate change and industrial competitiveness, most academics focus on the elements that put businesses at risk as a result of climate change. According to the available literature, the following was hypothesized.

*H_2_: Use of digital technology has led to the centralization of systems in organizations.*

### Role of Digital Technology Leading Transparency

New technology, the digitization of the prevailing processes and procedures, and a sustainable economic approach have altered business perspectives. In recent years, the terms “digitalization,” “information technology,” and “sustainable economy” have been the most widely used terms in literature (Attaran, 2021). If a company wants to stay competitive in the market, it must modify its approach to management. Emerging technologies have the potential to help in overcoming the development problems and support the objectives of universal coverage to most of the management services. Digitally transformed and digital enterprises promise a galaxy of apps and digitalized resources that combine to form new and innovative competencies that provide companies a competitive advantage. Latest technological adoptions are necessary for modernizing corporate processes such as administration, communication, and organization to make them more environmentally friendly.

The company must adapt and adjust its management technique in order to stay alive. In the digital era, digitalization and technological innovations for a green economy expose both potential and risks for employees and enterprises (Lee et al., 2016). Organization and management and cultural transformation are shaped by new technologies. An organization is a mechanism that individuals use to plan and organize their behaviors to gain what they want or value or to accomplish their objectives (Lei et al., 2021). The real implementation of the approach to business, though financial services or green fundamentals that have an immediate impact on employment and organizational structure, contends that: computer systems significantly decrease transaction fees, thus further trying to aid potential revenue, and also supports workers, purchasers, and community members in trying to challenge business practices; however, it may lead to the emergence to hacking attacks at about the same moment. The following was laid down as a hypothesis for the relation of technology with transparency.

*H_3_: Use of digital technology has led to transparency in organizations.*

### Moderating Role of Product Knowledge Hiding Between Digital Technology and Green Economy

The spread of internet misinformation and rumors has captivated our interest in recent years, since it has ideologically split society and resulted in social instability, weakened democracy, and other effects. Efforts to identify them on technological or behavioral aspects are widespread, but these efforts often fall short of addressing their origins, and so may not be able to prevent them from forming or spreading. The recent expansion of online news platforms has been fueled by the explosion of internet users (also known as “netizens”), while mainstream outlets have begun to leverage multiple internet resources, such as online media, for reporting. As a result, online information and news are now more widely disseminated and impactful. However, the internet has aided the spread of false information and fake news, deceiving readers and attracting widespread attention (Rochlin, 2017).

Product knowledge hiding can be defined as any incorrect information from an academic standpoint, whereas fake news relates to news stories that are purposely and indisputably untrue and deceptive. The phrase “fake news” is occasionally used to criticize media companies. Product knowledge hiding and fake news have a long history of deceiving readers, and it is not limited to the internet. Before the internet, there was the Great Lunar Conspiracy of 1835, which was an example of fake news. Even after the articles were proven to be a hoax, the New York Sun published pieces on the finding of life and civilization on the Moon, which resulted in a substantial increase in the newspaper’s circulation. Product knowledge hiding has evolved in tandem with technical advancements, particularly with the introduction of the internet. Emails containing urban legends and false warnings were widespread in the early 2000s. The growth of social media has simplified information sharing in recent years, blurring the lines between journalism and digital resources (Tandoc et al., 2018). On the other hand, it has made it easier to spread product knowledge hiding and fake news online, which has become a growing worry since it has the potential to sway public opinion on critical social problems.

Various facets of internet disinformation and fake news, such as their traits, enablers, incentives, and effects, are of interest to scholars (Greene et al., 2021). In fact, the majority of digital product knowledge hiding and fake news is mentally and tactically designed, sometimes using overstated language. Nonetheless, their sources and authors could be suspect. The motivations for creating and disseminating fake news and product knowledge hiding can be political or financial, and the dissemination of fake news and product knowledge hiding can be facilitated by a lack of trust in traditional media, audiences misled by misinformation/fake news, and various CMC (computer-mediated communication) technologies (Au et al., 2021). Researchers have been exploring several approaches to dealing with the effect of false information from both a technical and legal standpoint. However, each aspect may not be enough to solve the problem. Technology tools, for instance, may aid in the detection of fake news pieces and websites, but some consumers may choose to reject all misinformation detection methods. Regulation may limit the spread of internet misinformation including fake news with evil intentions, but it may not prevent some well-intentioned sharing that tries to help others (Tandoc et al., 2019).

People have access to an ever-increasing amount of knowledge *via* the media and internet. The access to information, on the other hand, does not appear to be linked to people’s improved knowledge. Information may be present, but it may not be well structured, organized, or even accessible. According to previous research, there are four main causes behind this: (1) much of the accessible information is false; (2) most of the available information is irrelevant; (3) information entropy: information is poorly organized and displayed; and (4) information overload: there is simply too much information for humans to comprehend (Farooq et al., 2020). Social networks can be seen to amplify both legitimate and fraudulent news. Facebook was the most popular social media platform in 2019, with over 1.5 billion registered users, and 62% of them were using it to keep up with news. Furthermore, studies frequently distinguish online websites from social media as a news source; yet, website news items are typically disseminated and shared exclusively through social media. As a result, social media is especially vulnerable to be utilized as a distribution tool for fake news. Almost half of those who share news (42%) admitted to spreading false information at some point.

There were instances of bots being used to spread fake news and efforts to detect fake news using algorithms. While bot systems that propagate bogus material can be identified and halted, algorithms cannot always tell the difference between fake and legitimate news. As a result, attempts to discover and filter phony news pieces on social media algorithmically will result in a huge number of false positives and false negatives, adding to censorship but yet allowing fake news to be broadcast. On the other hand, humans can make blunders in spotting bogus news, whether deliberately or unintentionally (Thompson, 2019). Aside from increasing the provision of reliable and well-structured knowledge and guiding people to it, there were various more recent scholarly proposals on reducing the detrimental effects of fake news and preventing people from propagating it. According to recent studies, people are more skeptical of online news if they have reason to believe the content is of poor quality. People who are prompted to pay attention to the source(s) of the news they are reading become more dubious of the knowledge and are less likely to spread fake news.

These findings suggest that social media platforms can affect people’s news sharing and reading skills. Another recent report recommended using crowd funding to fact-check and certify news stories’ legitimacy. In a sense, news article crowd sourcing is already in existence. Wikipedia, for example, might be considered a crowd-sourced knowledge repository. On the other hand, news items must be written and disseminated quickly, which necessitates speedy tools to predict fake news. The difficulty with most of the current recommendations for preventing the spread of disinformation is that they start to make a trade-off. While the quantity of fake news stories shared can be reduced, other undesirable implications, such as users’ constrained freedom, may begin to arise (Nekmat, 2020). Considering the role of product knowledge hiding in this digital technological world, we hypothesized that product knowledge hiding could play a moderating role between digital technology and reinforcing the green economy. Product knowledge hiding can have an effect on personal benefits including shopping, entertainment, and education. This may also play a moderating role towards organizational benefits including transparency, access to new markets, and centralization. It was also considered that product knowledge hiding may impact the environmental benefits, including cutting on energy, reducing pollutants, and reducing water consumption. Summing up all these factors, the following were hypothesized to evaluate the moderating impact of product knowledge hiding (Yingfei et al., 2021).

*H_4_: Product knowledge hiding moderates the relationship of digital technology and transparency in organizations.*

*H_5_: Product knowledge hiding moderates the relationship of digital technology and access of new markets by organizations.*

*H_6_: Product knowledge hiding moderates the relationship of digital technology and the centralization of systems in organizations.*

**Figure 1:**
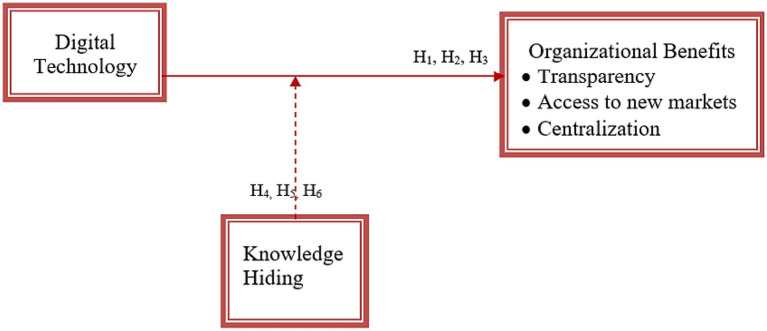
Conceptual framework. Central, Centralization; DT, Digital technology; NM, Access to new markets.

Based on the literature review, this research was designed. The following conceptual framework was developed (see Figure 1). The research revolves around this.

This framework has been hypothesized in light of the systems management theory. According to this theory, organizations and businesses work in multiple sub-systems. These should work in complete harmony and accordance with each other to get the maximum outcome from each complimentary sub-system. The success of any activity and business depends on the interdependence, synergy, and interrelations of each component with the other (Mele et al., 2010). In the present study, the framework has been devised on the concept of systems management theory that when the digital technology is utilized to gives so many interdependent and interrelated benefits which work the best with each other.

## Research Methodology

The current study has followed the positivist approach of research as it measures the causal effects of certain variables (Avotra et al., 2021a). This deductive approach has proposed different theories based on different hypotheses derived from the literature which would either be accepted or rejected on the basis of the analysis. The population of the study comprised working level managerial staff in the telecom industry in mainland China. This particular segment of people was chosen as the population for this study because these people are more directly involved in the online benefits of digital technology not only at the individual level but at the organizational level as well. This segment not only involves cultivating benefits but also hiding knowledge in the form of misinformation in gaining their individual, organizational, or environmental benefits. The sample taken from this population was 358 women based on the convenient random sampling because women are more involved in online shopping and entertainment along with organizational commitments. The data were collected at once in this cross-sectional study. Further, the data were analyzed using partial least square structural equation modeling (SEM) with the help of software Smart-PLS 3.3.

### Instrument Development

The questionnaire was comprised of 28 items in total. It was developed on the five-point Likert scale with 5 = strongly agree, 4 = agree, 3 = neutral, 2 = disagree, and 1 = strongly disagree. The items of use of digital technology (5 items), transparency (4 item scale), access to new markets (4 items scale), and centralization (3 items scale) have been adapted from Ciocoiu (2011). Moreover, the items of the variable knowledge hiding (12 item scale) have been taken from Connelly and Zweig (2015). Their scale of rationalized knowledge hiding was modified to compile the scale for measuring product knowledge hiding. The demographics included in the questionnaire involved the categories for age, education, and the length of service for the respondents. The analysis of demography on the basis of frequency and percentages is given in Table 1.

**Table 1:** Demographics of the respondents.

Age	Frequency	Percentage
<20	74	20.67
21–29	115	32.12
30–39	84	23.46
40–49	35	9.77
49>	50	13.96
**Education**		
Bachelor	80	22.34
Masters	189	52.79
Doctorate	65	18.15
Others	24	6.70
**Length of Service**		
<10	201	56.14
11–20	56	15.64
20>	101	28.21

N = 358.

## Data Analysis

**Figure 2:**
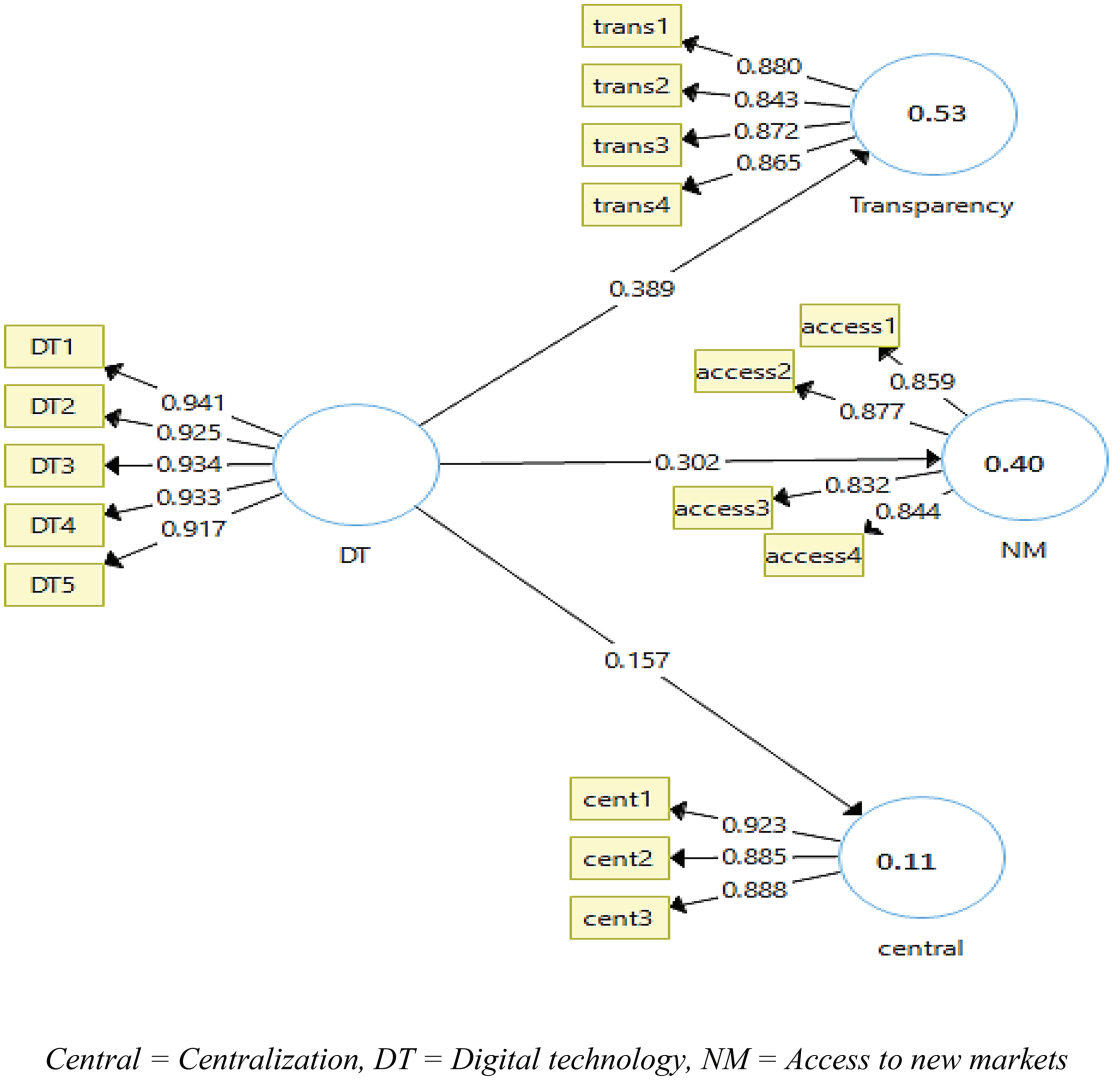
Measurement model algorithm. Central, Centralization; DT, Digital technology; NM, Access to new markets; KH, Knowledge hiding; TrpMod, Transparency moderation; NMMod, Access to new market moderation; Cent Mod, Centralization moderation.

Smart-PLS 3.3 was the software used for data analysis to measure the relationships among the hypotheses. The data in this study were analyzed in three stages. In the first stage, the demographic data in Table 1 were analyzed. In the second stage, the validity and the reliability of the data were measured with the help of the measurement models while in the third stage, the hypotheses were validated using the structural model with consistent bootstrapping of 500 iterations. The measurement model for this study can be seen in Figure 2.

On the basis of the measurement model, the reliability and validity of the data were checked. For the reliability, composite reliability and Cronbach Alpha reliability were used. Moreover, average variance extracted (AVE) was also used to check the validity. The reliabilities and AVE are reported in Table 2. The acceptable value mentioned in literature is above 0.7 for reliability (Avotra et al., 2021b). In the current study, the values of reliabilities for the independent, dependent, and moderating variables are above 0.7 for Alpha and composite reliabilities. The minimum value of Alpha reliability is 0.877 for the variable access to new markets while composite reliability is 0.915 which is among good values; hence, data are reliable in this study. As for the average variance extracted, the literature has mentioned the threshold of 0.5 (Hai et al., 2021). All the values in this study are above this threshold that can be seen in Table 2. The minimum value in this study for AVE is 0.658 which is for the variable access to new markets.

**Table 2:** Model measurements.

Constructs	Code	FD	*α*	CR	AVE
Access to New Market			**0.877**	**0.915**	**0.730**
	access1	0.838			
	access2	0.883			
	access3	0.843			
	access4	0.853			
Transparency			**0.888**	**0.923**	**0.749**
	trans1	0.883			
	trans2	0.838			
	trans3	0.875			
	trans4	0.865			
Centralization			**0.883**	**0.928**	**0.811**
	cent1	0.903			
	cent2	0.898			
	cent3	0.901			
Digital Technology			**0.961**	**0.970**	**0.865**
	DT1	0.941			
	DT2	0.925			
	DT3	0.934			
	DT4	0.933			
	DT5	0.917			
Knowledge Hiding			**0.952**	**0.958**	**0.658**
	KH1	0.778			
	KH2	0.810			
	KH3	0.809			
	KH4	0.809			
	KH5	0.856			
	KH6	0.878			
	KH7	0.886			
	KH8	0.875			
	KH9	0.764			
	KH10	0.743			
	KH11	0.754			
	KH12	0.758			

*N = 358, FD = Factor loading, AVE = Average variance extracted, CR = Composite reliability. Bold mean optimal values.*

The data were additionally validated with Fornell and Larcker criterion and HTMT (Heterotrait-Monotrait) ratio. According to literature, for data to be valid, the values of Fornell and Larcker criterion should be higher in each column at the top than the rest of the column (Henseler et al., 2015; Franke and Sarstedt, 2019). All the values in this study for Fornell and Larcker criterion meet this criterion as all values at the top of each column are the highest. The results can be seen in Table 3.

**Table 3:** Fornell and Larcker criterion.

	DT	KH	NM	TRP	CENT
DT	**0.930**				
Kh	−0.310	**0.811**			
NM	0.298	−0.609	**0.854**		
TRP	0.389	−0.689	0.658	**0.865**	
CENT	0.153	−0.332	0.512	0.513	**0.900**

*N = 358, CNT = Centralization, DT = Digital technology, NM = Access to new markets, TRP = Transparency, KH = Knowledge hiding. Bold mean optimal values.*

Similarly, the HTMT ratio needs to be significant if the values of the table are much less than 0.9 (Henseler et al., 2015; Franke and Sarstedt, 2019). All the values in this study for the HTMT ratio table are less than 0.90 which show that the variables are different yet valid for measuring their own unique constructs. The results can be seen in Table 4 of the study.

**Table 4:** HTMT ratio.

	DT	KH	NM	TRP	CENT
DT					
Kh	0.326				
NM	0.320	0.656			
TRP	0.420	0.747	0.741		
CENT	0.164	0.361	0.591	0.580	

*N = 358, CNT = Centralization, DT = Digital Technology, NM = Access to New Markets, TRP = Transparency, KH = Knowledge hiding.*

The r-square values obtained above show that the variables selected for the study have been strong enough to be studied. The highest level of strength of a model is shown by the variables transparency (R-square value = 53.7%) and access to new markets (R-square value = 40%), while centralization showed an 11% value for R-square. These values show the strength of the model and significance of these variables in this framework. F-statistics obtained from the results also support the results of t-statistics. Transparency (F-square = 0.17%) and access to new markets (F-square = 0.100) have been found to be very significant factors for the benefit of using digital technology as their values are showing a very large effect (Sureiman and Mangera, 2020). Moreover, centralization shows a very small effect.

**Figure 3:**
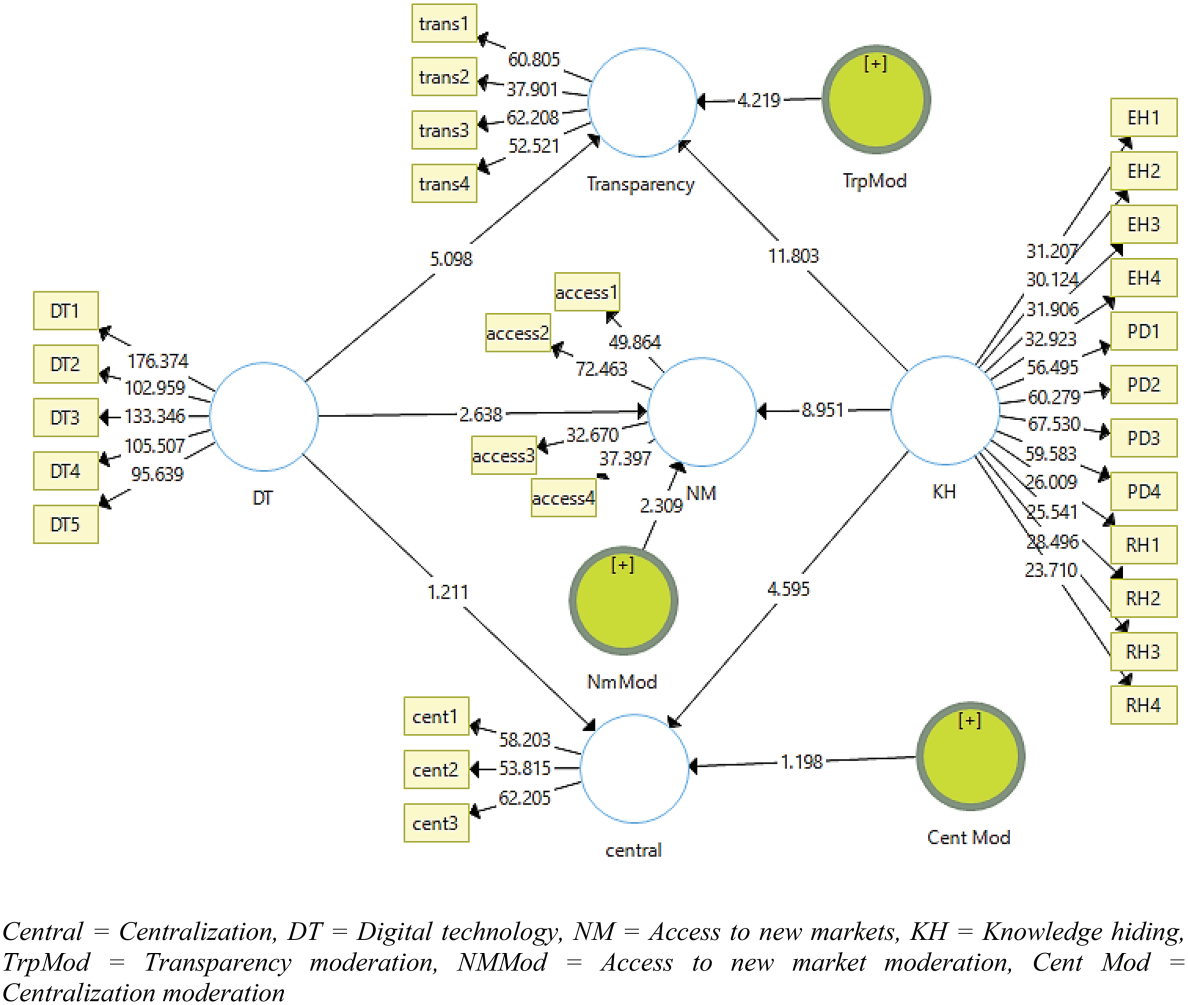
Graphical model for bootstrapping algorithm.

The third stage of the data analysis was the interpretation of the structural model algorithm obtained by consistent bootstrapping. The results of the algorithm can be seen in Figure 3. In this phase, the hypotheses developed in the study were checked to determine whether they were supported by the data or not. This study used beta values, *t*-statistics, values of *p*, R-square, and f-square to check the acceptability of the hypotheses. The results can be seen in Figure 3 and Tables 5, 6.

**Table 5:** Results for structural model.

Paths	H	O	M	SD	T-Stats	P-Value	Results
DT →TRP	H_1_	0.200	0.203	0.039	5.098	0.000^***^	*Accepted*
DT →NM	H_2_	0.125	0.130	0.048	2.638	0.009^**^	*Accepted*
DT →CNT	H_3_	0.058	0.062	0.048	1.211	0.226^*^	Rejected

N = 358, H = Hypotheses, O = Original sample, M = Sample mean, SD = Standard deviation, ***p < 0.0005, **p < 0.005, *p < 0.5, CNT = Centralization, DT = Digital technology, NM = Access to new markets, TRP = Transparency.

**Table 6:** Moderation effect.

Paths	H	O	M	SD	T-Stats	Value of *p*	Results
TrpMod →TRP	H_4_	0.158	0.159	0.038	4.219	0.000^***^	*Accepted*
NM Mod→NM	H_5_	0.117	0.120	0.051	2.309	0.021^*^	*Accepted*
CNTMod →CNT	H_6_	0.066	0.075	0.055	1.198	0.002^**^	Rejected

N = 358, H = Hypotheses, O = Original sample, M = Sample mean, SD = Standard deviation, ***p < 0.0005, **p < 0.005, *p < 0.5, TrpMod = Transparency moderation, NMMod = Access to new market moderation, Cent Mod = Centralization moderation, CNT = Centralization, NM = Access to new markets, TRP = Transparency.

Table 5 gives the results of consistent bootstrapping for the rejection or acceptance of the hypotheses developed in the literature. In total, there were 18 hypotheses developed in this study segregated as individual, organizational, and environmental benefits of the use of digital technology. Among each category there were a further three hypotheses. The first hypothesis (H_1_) addressing transparency among three benefits for the organizations was found significant with t−statistic=5.098:p−value=0.000∗∗∗ , and access to new markets (H2) with t−statistic=2.638:p−value=0.009∗∗ was accepted at a 0.1% significance level thus hypotheses H_1_ and H2 were accepted. Furthermore, centralization found no contribution from the use of digital technology ( t−statistic=1.211:p−value=0.226 ), thus H_3_ was rejected at a 5% level of significance.

Proceeding to the moderation of product knowledge hiding in organizational benefit; it significantly moderates the relationship of use of digital technology and transparency ( t−statistic=4.219:p−value=0.000 ), therefore H_4_ was accepted_._ Moreover, H5 found a moderation of access to new markets with t−statistic=2.309:p−value=0.021 accepting H_5;_ along with the rejection of moderation to centralization t−statistic=1.198:p−value=0.232 , thus rejecting H_6_. Table 6 shows the r-square values obtained for the variables in the current study.

## Discussion

This research was based on several hypotheses to analyze benefits of digital technology on reinforcing the green economy based on the analysis of several factors including personal, organizational, and environmental benefits. Product knowledge hiding in the digital era has a great potential to be explored and was analyzed for its moderating role in this study. The data in this study were analyzed in three stages. In the first stage, the demographic sheet was analyzed. In the second stage, the validity and the reliability of the data were measured with the help of the measurement models, while in the third stage, the hypotheses were validated using the structural model. A theoretical framework was designed and questionnaires were sent to managerial staff of the telecom sector in China. The results mostly supported the hypotheses. The results were also in accordance with many researchers, but some were of the different opinion. The possible reasoning for the obtained results is also discussed here. All participants had different education levels ranging from Bachelors to Doctorate level.

The cut off values for reliability is said to be 0.7 (Avotra et al., 2021b). All the values in this study are above 0.70 ranging from 0.877 to 0.961 for Alpha reliability. Hence the data in this study are reliable. As for the average variance extracted, the literature has mentioned the threshold of 0.5 (Hai et al., 2021). All the values in this study are above this threshold that can be seen in Table 2. The minimum value in this study for AVE is 0.658 which is for the variable access to new market. The possible reason for getting these results was the authenticity and reliability of the data collected from the participants. According to literature, for data to be valid, the Fornell and Larcker criterion values should be higher in each column at the top than the rest of the column (Henseler et al., 2015; Franke and Sarstedt, 2019). All the values in this study for the Fornell and LArcker criterion meet this criterion as all values at the top of each column are the highest. Similarly, the HTMT ratio needs to be significant if the values of the table are well below 0.9 (Henseler et al., 2015; Franke and Sarstedt, 2019). All the values in this study for the HTMT ratio are well below 0.90, showing that the variables are different yet valid for measuring their own unique constructs. The third stage of the data analysis was the interpretation of the structural model algorithm obtained by consistent bootstrapping. This is usually the subsequent stage of the measurement model.

In total, six hypotheses were developed regarding organizational benefits of the use of digital technology. Moving to the organizational benefit of the use of digital technology, among three benefits transparency, centralization, and access to new markets were accepted thus accepting hypotheses H1, H_2,_ and H_3_. These kinds of results were obtained due to the possible reason of organizational structure, as digital technology has reduced the malpractices of corrupt individuals and transparency and access to new markets have become easy (Moşteanu et al., 2020). The reason behind it is the flow of correct information. The results of the study reject the moderation of centralization, rejecting H_6._ However, transparency and access to new markets was found to be moderated from knowledge hiding, since transparency itself is very important with fair availability of information and any hidden knowledge can be costly (Farooq et al., 2020). The reason for obtaining such results is that digital technologies have certain benefits regarding reinforcement of the green economy, but knowledge hiding may lead to deterioration of the objective of obtaining information for digital use of technology (Xiaolong et al., 2021).

## Conclusion

Digital technology has been the center of attention for a while for researchers. It has led to different findings and created new horizons relating to digital entrepreneurship, digital economies, etc. The initiation of digitalization leads to infinite opportunities to reap different benefits not only at the individual level but also at the mass level of organization and environment. This current study is also one such effort to explore the unprecedented diverse benefits of digitalization and use of digital technology. The study has found a significant role of digital technology in molding organizational activities adhering them according to the emerging demands of incorporation of new technologies. At the organizational level, transparency in the organizations, access to new markets, and centralization has been majorly affected by the use of digital technology. However, like any other progress, this also brings in the challenges of product knowledge hiding or overflow of information with it that interrupts the steep flow of big data. The main focus of this study has been on the understanding of the benefits of use of digital technology organizations by the women of the telecom sector in China. After conducting this cross sectional research, the results have shown that there are certain organizational benefits in the use of digital technology. The current study has a certain implication in real life due to the emergence of new technologies; individuals are indulging in online activities. Hence the policies of the organizations should be made according to how customers can be catered to online regarding shopping and education. Secondly, organizations can also imply the use of digital technology to centralize working operations and hence make access to new markets possible by optimally exploiting the use of technology in business.

### Theoretical Contributions

The study has theoretical contributions, such as (1) the present study contributes to expanding the network of knowledge hiding, addressing the moderation of product knowledge hiding, and extending the known consequences of digital technology influencing knowledge hiding behavior, (2) the role of knowledge hiding in the online world, especially in the post-COVID scenario, has changed dramatically. Hence, the need to save online interactions from the impact of knowledge hiding is urgently needed, and (3) this study adds to the body of literature regarding knowledge hiding by finding its implications for organizations in dealing with off-shore partners.

### Practical Implications

The study has practical implications, such as (1) this research has several implications for future researchers and policymakers for reinforcing the advantage of digital technology for organizations, (2) organizations, especially off-shore companies can make their policies and can access new markets by utilizing digital technology and ensuring the detainment of knowledge hiding by the personnel employed in handling these departments, (3) it is also an opportunity for researchers who are interested in repeating this research with their available resources in different regions and by adopting different factors. These can be exploited well in finding new avenues for certain studies like this.

### Limitations and Future Contributions

This research faced certain limitations which could be rectified in the coming projects. Some of these limitations include over-dependency on digital technologies, the huge costs of using the latest digital technologies, risk of job loss, extra favor of online businesses causing store closures, data security issues, etc. Other limitations of the study include the amount of time, which was quite small, as shifting to a green economy requires a lot of time. Overcoming the main issue of time will surely result in a worthy outcome. In future, working on similar research, these findings of the present study should be kept in mind. It is also an opportunity for researchers who want to expand this research with their available resources in different regions and are interested in adopting different factors. These can be exploited well in finding new avenues for certain studies like this.

## Data Availability Statement

The original contributions presented in the study are included in the article/supplementary material, further inquiries can be directed to the corresponding author.

## Ethics Statement

All subjects gave their informed consent for inclusion before they participated in the study. The study was conducted in accordance with the Declaration of Helsinki, and the protocol was approved by the Southeast University, China.

## Author Contributions

The author confirms being the sole contributor of this work and has approved it for publication.

## Funding

This research received no external funding and the study was supported by Southeast University, China.

## Conflict of Interest

The author declares that the research was conducted in the absence of any commercial or financial relationships that could be construed as a potential conflict of interest.

## Publisher’s Note

All claims expressed in this article are solely those of the authors and do not necessarily represent those of their affiliated organizations, or those of the publisher, the editors and the reviewers. Any product that may be evaluated in this article, or claim that may be made by its manufacturer, is not guaranteed or endorsed by the publisher.
